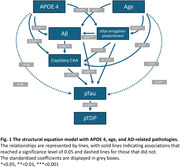# Connection between APOE4 and tau is mediated via amyloid‐beta and Alzheimer’s co‐pathologies in human autopsy brains

**DOI:** 10.1002/alz.086811

**Published:** 2025-01-03

**Authors:** Klara Gawor, Sam Verrept, Celeste Laureyssen, Sandra O. Tomé, Rik Vandenberghe, Mathieu Vandenbulcke, Philip Van Damme, Christina von Arnim, Markus Otto, Estifanos Ghebremedhin, Alicja Ronisz, Simona Ospitalieri, Matthew Blaschko, Jasper Van Dongen, Kristel Sleegers, Dietmar Rudolf Thal

**Affiliations:** ^1^ Leuven Brain Institute, Leuven Belgium; ^2^ Laboratory for Neuropathology, KU Leuven, Leuven Belgium; ^3^ UZ Leuven, Leuven Belgium; ^4^ Department of Biomedical Sciences, University of Antwerp, Antwerp Belgium; ^5^ Complex Genetics of Alzheimer’s Disease Group, VIB Center for Molecular Neurology, VIB, Antwerp Belgium; ^6^ Department of Neurosciences, KU Leuven, Leuven Belgium; ^7^ Neuropsychiatry unit, KU Leuven, Leuven Belgium; ^8^ University of Ulm, Ulm Germany; ^9^ University Medical Center Göttingen, Göttingen Germany; ^10^ University Hospital Ulm, Ulm Germany; ^11^ Goethe University Frankfurt, Frankfurt Germany; ^12^ Center for Processing Speech & Images, KU Leuven, Leuven Belgium; ^13^ Complex Genetics of Alzheimer’s Disease group, VIB‐UAntwerp Center for Molecular Neurology, Antwerp Belgium; ^14^ University of Antwerp, Antwerp Belgium

## Abstract

**Background:**

Alzheimer’s disease (AD) brains commonly exhibit various co‐morbid pathologies, with cerebral amyloid angiopathy (CAA) being the most prevalent, affecting 70‐90% of patients. CAA can be restricted to medium and large vessels or extend to capillaries. Additionally, AD patients often show pathologies involving phosphorylated‐TDP‐43 (pTDP‐43) and alpha‐synuclein (αSyn), typically demonstrating an amygdala‐predominant subtype. The apolipoprotein E ε4 allele (APOE ε4) is a major genetic risk factor for Amyloid Beta (Aβ) pathology, but uncertainties remain regarding its impact on phosphorylated‐tau (pTau) and pTDP‐43.

**Methods:**

To address this knowledge gap, we conducted a neuropathological examination of 237 brains, including 89 AD patients. We assessed pTau stages, Aβ phases, capillary involvement in CAA, and the presence of pTDP‐43 in the hippocampus. We also examined αSyn in the medulla oblongata, identifying the amygdala‐predominant variant using additional staining. APOE genotyping was performed on all individuals from frozen or paraffin‐embedded brain tissue.

**Results:**

Our structural‐equation model revealed interrelationships among AD‐related neuropathologies, APOE, and age. We confirmed previous findings on substantial influence of the APOE ε4 allele on Aβ levels. We also identified an equally robust effect on capillary CAA, even after adjusting for the impact of Aβ on this pathology. Amygdala‐predominant αSyn was also affected by the presence of an APOE ε4 allele. Significant associations were observed between pTau and all measured pathologies. Importantly, after accounting for interactions, APOE ε4 showed no effect on pTau and pTDP‐43.

**Conclusions:**

Our study suggests that APOE ε4 primary influence on pathological Tau is likely mediated through associations with Aβ, capillary CAA, and amygdala‐predominant αSyn. Similar conclusions might hold for APOE ε4 and p‐TDP‐43, although further confirmation with a larger sample size is needed. Despite indications from animal models, our results suggest that the impact of APOE ε4 on pTau in the human brain may be minimal, linked to its high interconnection with other brain pathologies.